# Revealing environmental synchronicity that enhances anchovy recruitment in the Mediterranean Sea

**DOI:** 10.1038/s41598-022-09418-z

**Published:** 2022-04-04

**Authors:** F. Quattrocchi, G. Garofalo

**Affiliations:** National Research Council - Institute for Marine Biological Resources and Biotechnology (CNR IRBIM), SS Mazara del Vallo, Via L. Vaccara 61, 91026 Mazara del Vallo, TP Italy

**Keywords:** Fisheries, Marine biology, Climate-change ecology

## Abstract

Small pelagic fishes in the Mediterranean Sea constitute about half of the total landings, of which almost one-third is European anchovy. Anchovy abundance mainly depends on early life stage and juvenile survival and growth, which are susceptible to shifts in environmental processes. Due to the commercial importance of this species, it is necessary to elucidate the processes affecting recruitment strength for effective fishery management, using environmental indices to set more appropriate harvesting limits. Here, we constructed a simple index to capture synchronicity between enrichment and retention/concentration processes, which are known to affect anchovy abundance, during the first year of life. Three ecosystems in the Mediterranean were examined: Gulf of Lions, Adriatic Sea, and Strait of Sicily. The synchronicity index (SI) represented the synergic evolution over time of the chlorophyll-a concentration (CHL, enrichment process) and mixed layer depth (MLD, concentration/retention processes), and was related with the abundance of anchovy recruits obtained from published survey reports. Considering different ecosystems, when the SI was significantly higher, anchovy recruitment was promoted. This result indicated SI is consistent across ecosystems in explaining anchovy abundance fluctuations and thus could be used to enhance fisheries management and extended to assess the impact of projected environmental changes.

## Introduction

The global distribution of small pelagic fishes, their high biomass at the mid-trophic level of food webs, and the role they play linking lower and higher trophic levels make them an essential component of marine ecosystems^1,2^. In the Mediterranean Sea, small pelagic fishes are targeted by both artisanal and industrial fisheries, and represent about 44.3% of total landings (from 2016 to 2018^3^). These species are characterized by short life spans, fast growth, early onset of maturity, and direct dependence on plankton, making them particularly sensitive to the environment e.g.^4^. Their abundance is highly dependent on a successful annual recruitment. In turn, early life stages (ELS) of small pelagic fishes are extremely susceptible to shifts in physical and biological processes influencing their survival^5–7^ because their tolerance ranges for various abiotic and biotic factors are narrower than those of adults^8^. Consequently, environmental fluctuations cause variation in the abundance and biomass of small pelagic fish populations^4^. This phenomenon can have substantial consequences for both fisheries and ecosystem structure e.g.^1^.

European anchovy (*Engraulis encrasicolus*) is an important fishery resource that represents 14.1% of total catches in the Mediterranean basin (from 2016 to 2018^3^). This species reaches maturity during the first year of life^9^. It reproduces from April to October^10,11^. Due to the commercial importance of this species in the Mediterranean Sea, an extensive number of studies have primarily focused on understanding how environmental factors contribute to its irregular fluctuations in abundance (e.g. Fernández-Corredor et al*.*^12^ and references therein). Most studies identified the key roles of different exogenous factors (mainly related to spatiotemporal food availability) in affecting recruitment strength by acting on growth and survival during ELS. Studies have assessed deviations from or matches with well-established hypotheses on processes related to the recruitment success of anchovy. These hypotheses include: (1) the link between recruitment strength and spatiotemporal overlap of enrichment, concentration, and retention processes (ocean triads hypothesis^5,13^); (2) the importance of calm ocean conditions and stratification of the water column following enrichment events, which favor plankton aggregation and thus ELS provisioning (stable ocean hypothesis^14^); and (3) the importance of spatiotemporal overlap between the abundance of ELS and their prey in promoting recruitment (match–mismatch hypothesis^15^).

The physical mechanisms promoting these three hypothesized processes differ among areas where anchovy occur and reproduce in the Mediterranean. In the Gulf of Lions (north-western Mediterranean basin), for example, upwellings and river outflow contribute to enrichment, with wind-induced downwellings promoting concentration and retention. In the Adriatic Sea (north-eastern part of the central Mediterranean basin), strong freshwater inputs promote the high enrichment of nutrients, with large areas of low turbulence and strong stratification in summer promoting concentration and retention processes^13^. In contrast, in the Strait of Sicily (central Mediterranean Sea linking the east and west basins), favorable conditions depend almost exclusively on circulation patterns, which are dominated by the interaction of the Atlantic-Ionian Stream (AIS) and bottom topography^16^. These differences in physical mechanisms generate distinct, and sometimes contrasting, relationships when linking ELS and juvenile abundance estimates with environmental factors, which are often used as proxies for processes affecting recruitment strength.

For example, chlorophyll-a (CHL) concentration (a proxy for primary productivity e.g.^17^) is not linked to the abundance of anchovy eggs in the Strait of Sicily^18^, whereas a negative (but not significant) correlation was found in the Adriatic Sea^19^, and a non-linear correlation was found in the western Mediterranean Sea where intermediate productivity favored egg abundance^17^. Furthermore, temperature, which acts directly on growth and is used as a proxy of water column stability, had a nonlinear influence on the density of anchovy eggs and larvae in the northwestern Mediterranean with a positive effect only within a specific range (20–25 °C), but a negative effect in the Adriatic Sea and Strait of Sicily^17,18,20^. Similar discrepancies among subregions of the Mediterranean basin have arisen in studies focusing on juvenile and adult anchovy (see Fernández-Corredor et al*.*^12^ and references therein). Thus, elucidating the processes regulating recruitment strength could help improve fishery management, which needs to incorporate environmental indices to establish reasonable limits for harvesting target species^21,22^. Yet, these environmental indices and their effects are often specific in space and time; consequently, they cannot be applied across populations. This is the case for anchovies in the Mediterranean Sea, which are well-adapted to the varying environmental features in which they occur.

**Figure 1:**
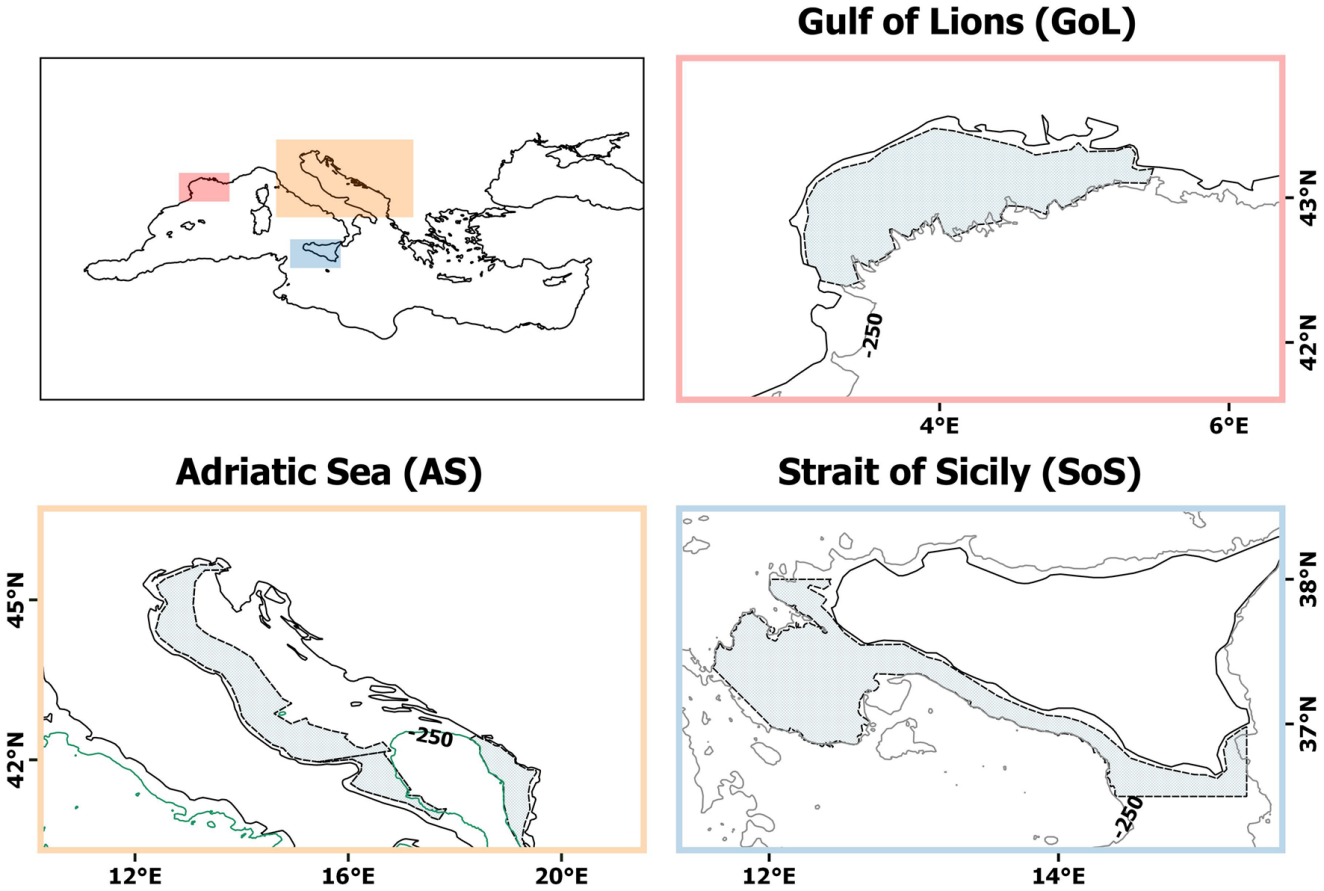
Study areas (solid bathymetric line = 250 m depth). Polygon symbols in the lattice indicate the three areas surveyed by MEDIAS (Strait of Sicily and Adriatic Sea) and PELMED (Gulf of Lions). QGIS software v. 2.18.28 was used to produce the map (QGIS Development Team. QGIS Geographic Information System. Open Source Geospatial Foundation Project; 2019. Available from: http://qgis.osgeo.org).

Here, a simple index was formulated that captured the processes known to influence the ELS and juveniles of anchovy, and so recruitment to the adult population, overcoming region-specific differences in the intrinsic environmental conditions where the species occurs. The index captures synchronicity between enrichment and retention/concentration processes during the first year of life of anchovy; consequently, it encapsulates conditions during the critical period of survival and growth before individuals enter the adult population. Specifically, the synchronicity of these processes reflects the synergic evolution of CHL concentration (enrichment process) and mixed layer depth (concentration and retention processes) during the temporal window running from the beginning of the spawning period until recruits are 1 year old. One year is used as a proxy of recruitment for anchovy, being the age at which individuals reach maturity^23^ and enter the fishery e.g.^24,25^. Although anchovy are adapted to different geographical areas with specific physical and biological factors, a higher index during ELS and juvenile stages would lead to higher anchovy recruitment (abundance of 1-year-old individuals), independent of specific environmental conditions. Abundance information was obtained from acoustic surveys carried out in the three Mediterranean Sea areas identified above; namely, the Strait of Sicily, Gulf of Lions, and Adriatic Sea. We standardized the data from the different regions to obtain a unique dataset of Age 1 anchovy abundance. The aim of our work was therefore to analyze the relationship between the synchronicity index and the SST and the recruitment of anchovy within the Mediterranean basin. The results of this study are expected to advance our understanding of how environmental variability drives the population dynamics of European anchovy in the Mediterranean Sea while providing an approach that could be easily applied to other species and systems (Fig. 1).

## Results

**Figure 2:**
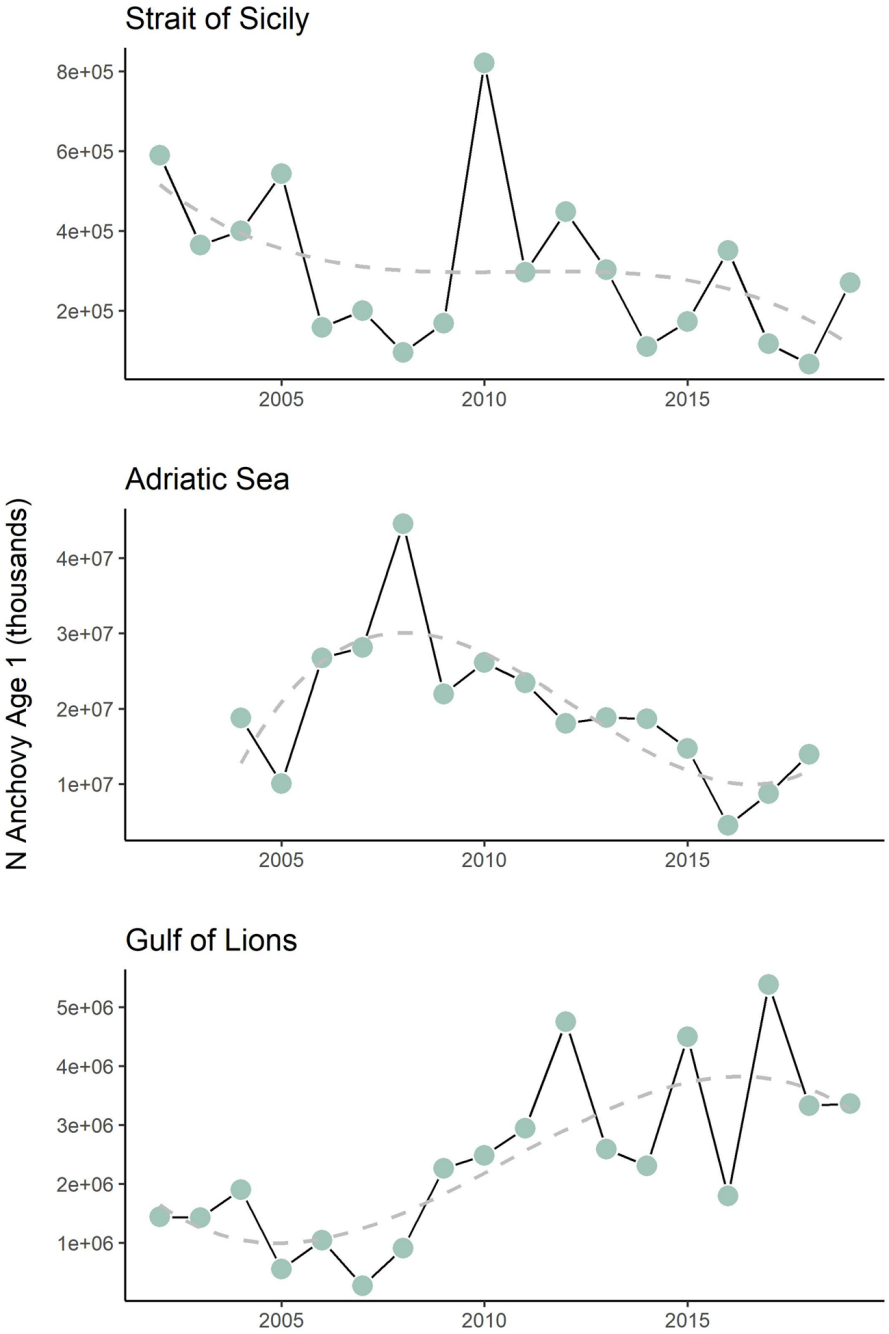
Age 1 anchovy acoustic abundance and trends estimated from the pelagic MEDIAS surveys (Strait of Sicily and Adriatic Sea) and PELMED (Gulf of Lions).

Total anchovy recruitment of the three areas are reported in Fig. 2. In the Gol anchovy Age 1 abundance increased during the period considered and it was at the lowest value in 2007 (ca. 2 + e05 thousands individuals) and at the highest in 2017 (5e + 06 thousands individuals) (Fig. 2). (Fig. 2). For AS, in which the abundance showed a decreasing trend, the highest and lowest anchovy recruitment was recorded in 2008 (ca. 4 + e07 thousands individuals ) and 2016(Fig. 2)(ca.4 + e06 thousands individuals ). In the SoS, the highest and lowest anchovy recruitment was recorded in 2018 (ca. 6 + e04 thousand individuals) and 2010 respectively, (ca8 + e05 thousand individuals). and it declined over time (Fig. 2).

**Figure 3:**
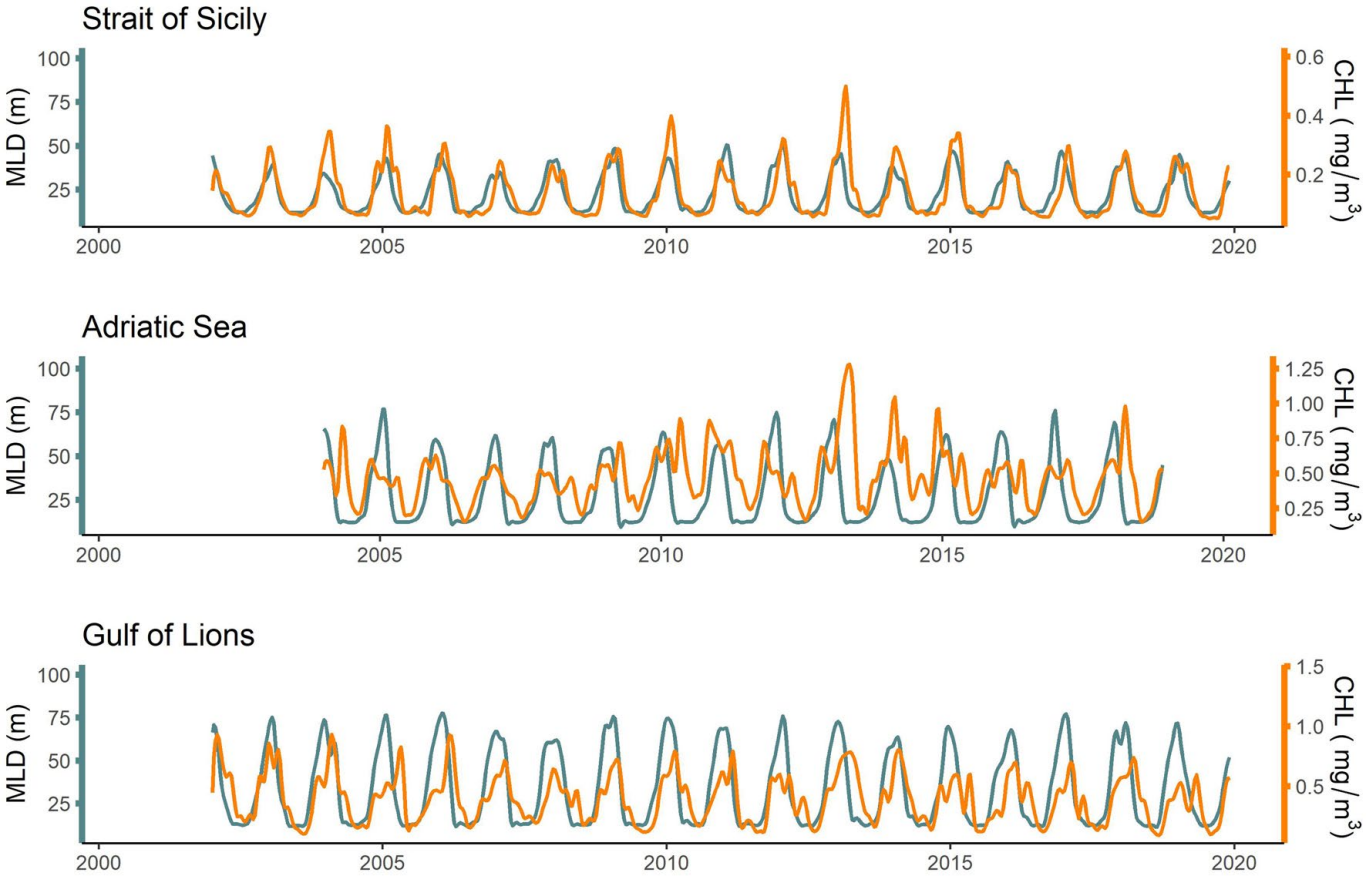
Fluctuations in chlorophyll-a concentration (CHL) and mixed layer depth (MLD) among the three surveyed ecosystems (Gulf of Lions, Adriatic Sea, and the Strait of Sicily).

On the continental shelf, the MLD of the SoS ranged between ca. 12 to 50 m depth, whereas it had a range of 12–76 m and 12–72 m in the GoL and AS, respectively (Fig. 3). CHL concentrations were highest in the AS (range: 0.16 to 1.28 mg/m^3^), followed by the GoL (range: 0.09 to 0.93 mg/m^3^) and the SoS (0.05 to 0.49 mg/m^3^), with the maximum value of the latter being 53% and 38% lower compared to the AS and GoL, respectively (Fig. 3).

**Figure 4:**
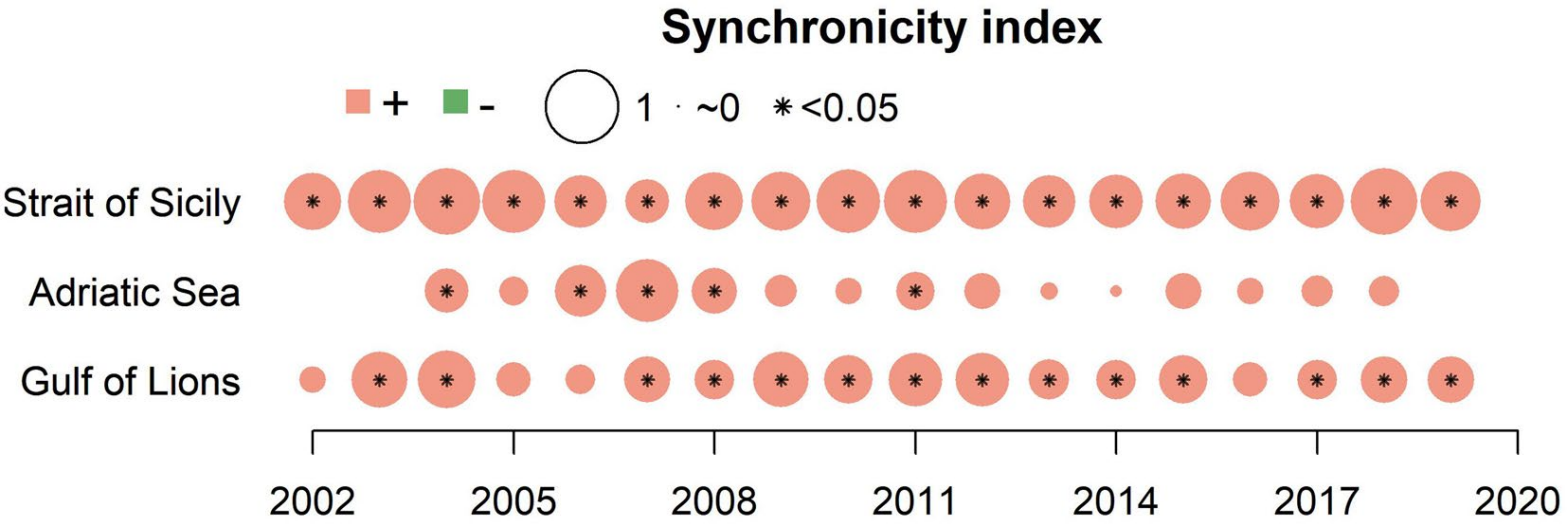
Synchronicity Index for each year and ecosystem. This was calculated by correlating CHL and MLD from the month corresponding to the beginning of the spawning season until the month preceding MEDIAS and PELMED observations the year after. Colors represent the sign of the correlation coefficient (pink = positive, green = negative). Point dimension represents the magnitude of the correlation coefficient. (*) indicates a significant correlation.

In all three areas, the synchronicity index (SI) indicated that MLD and CHL consistently co-varied positively over time. In the SoS, the SI was on average 0.80 ± 0.08 sd, and remained significant throughout the study period (Fig. 4). In the GoL, the SI was on average 0.61 ± 0.13 sd, and was significant for values above 0.5. In the AS, MLD and CHL co-varied significantly in 2004, between 2006 and 2008 and in 2011. During the other years, the SI was below 0.5 (range: 0.18–0.48),and was not significant (Fig. 4).

During the first year of life of anchovy, the SST was, on average, highest in the SoS, and increased over time in both the SoS and AS; however, no clear trend was detected in the GoL (Fig S1).

**Figure 5:**
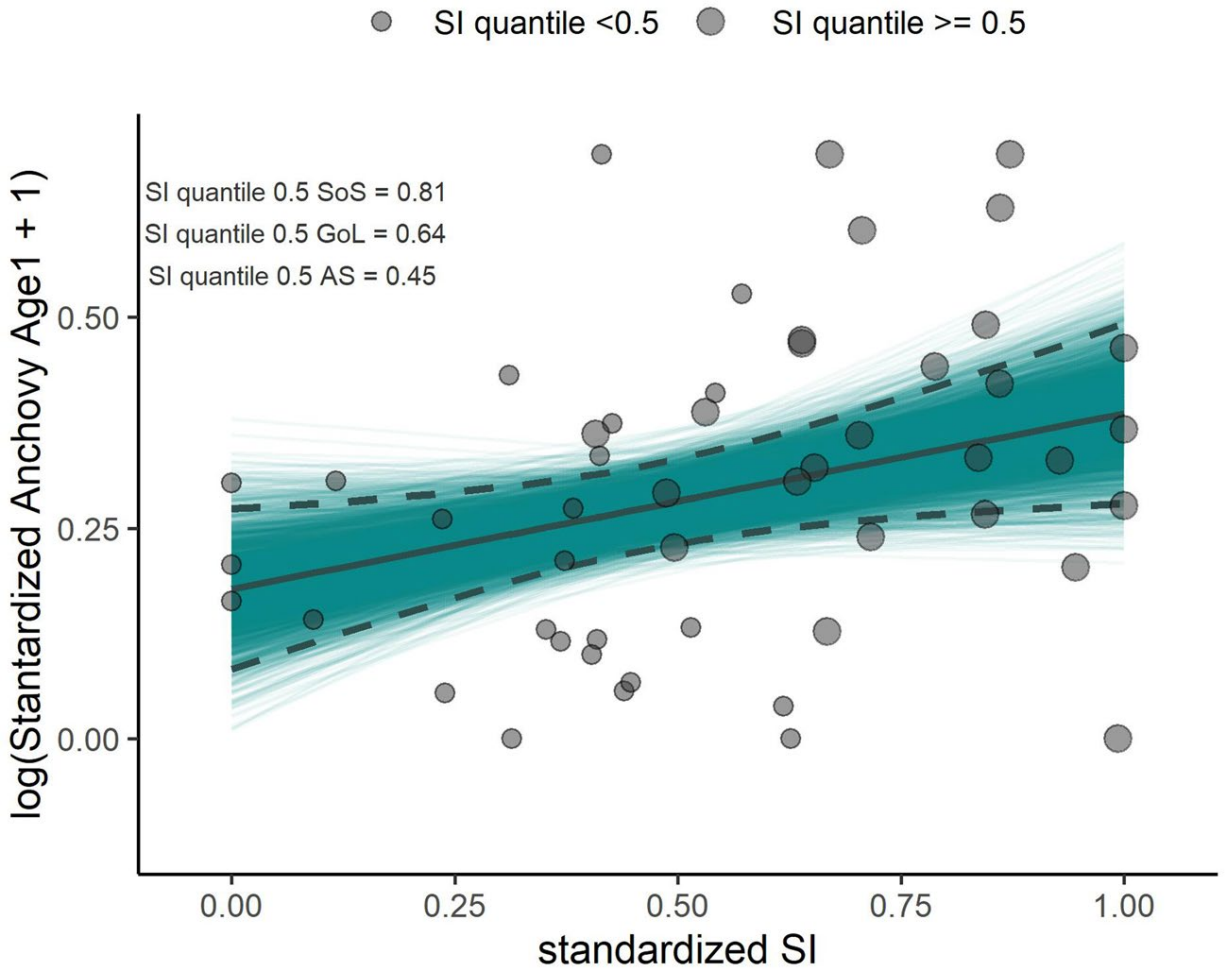
Standardized anchovy recruits log (x + 1) transformed as a function of the standardized synchronicity index. Fitted curve (solid line) and the 95% of confidence interval (dashed lines) of the n = 2999 bootstrapped fitted curves (blue lines). Point dimension represent values of SI of each area greater or less of its median value .

The robust regression model applied on the whole standardized dataset showed a positive effect of the SI on the anchovy recruitment and it was significant as showed by the confidence intervals obtained with the bootstrap resampling procedure. Contrarily, no significant effect of temperature was observed (Fig. 5, Table 1).

**Table 1:** Results of the Robust regression model (rlm). The covariates included are the Synchronicity Index (SI) and sea surface temperature (SST). The significance of the parameters (Intercept and slope) are obtained by calculating the 95% of confidence interval (CI) from the random x-resampling procedure. These CIs are the first order normal approximation (Normal), the bootstrap percentile interval (Percentile), and the adjusted bootstrap percentile (BCa).

	rlm parameters	CI 95% normal	CI 95% percentile	CI 95% BCa
Mediterranean Sea (SoS + AS + Gol)	*Intercept* 0.21	[0.06, 0.34]	[0.09, 0.37]	[0.09, 0.36]
	*Slope* SI = 0.21	[0.03, 0.38]	[0.03, 0.39]	[0.03, 0.39]
	SST = −0.07^ ns^	[−0.22, 0.09]	[−0.24, 0.09]	[−0.23, 0.09]

## Discussion

This study provides a step towards elucidating how environmental variability drives fluctuations in the abundance of anchovy populations occupying different ecosystems in the Mediterranean Sea. Through formulating a synchronicity index that captured covariation in mixed layer depth and chlorophyll, it was possible to delineate how the timing of key events in marine ecosystems impacts the critical period of anchovy growth and survival (from eggs through larvae to 1-year-old individuals). We showed that high synchrony between proxies of enrichment (CHL) and concentration/retention (MLD) processes during the first year of life increased the abundance of recruits entering the adult population.

Furthermore, contrary to expectations, our results indicated that temperature negatively affects the abundance of anchovy recruits but the effect is not significant, and thus temperature is not a potential driver of interannual fluctuations in anchovy recruitment during the evaluated period. Temperature is an essential climate variable^26^ and one of the bottom-up controllers for anchovy populations^4^. This lack of correlation with temperature might be explained by the average temperature values (i.e., from 15 to 25 °C) observed in each region falling within the general optimal temperature ranges that characterize the spawning habitat in which anchovy ELS are found in the Mediterranean Sea^17,24,27^. However, significant negative effects on anchovy ELS tend to arise at extremely high temperatures^12,17^. In the present study, these high values were not reached, although the temperature used here was the average of the whole period in which anchovy hatch and grow, hence masking the high values occurring during the hot seasons.

The synchronicity index developed here captures two key environmental processes that promote recruitment success through integrating temporal covariation in MLD and CHL. In mid-spring, when the mixed layer depth begins to shallow, high CHL concentrations occur, which decline in summer due to the MLD-mediated abiotic control (inhibiting flux in nutrients toward the surface) and zooplankton-mediated biotic control (Lavigne et al*.*, 2013 and references therein^28^). Because zooplankton are the main prey of anchovy e.g.^29,30^, increased concentrations of zooplankton facilitate the survival and growth of anchovy larvae in spring and summer. The spawning period of anchovy matches these optimal conditions, allowing young individuals to feed and accumulate energy during the reproductive period (income breeding strategy^31^). Furthermore, high synchrony during late fall-winter reflects the beginning of upward mixing (the MLD deepening), which allows nutrients to increase and raise CHL biomass^19,32^, facilitating the survival of pre-recruits. High synchrony provides better environmental conditions, enhancing growth and allowing anchovy juveniles to achieve safe overwintering size, ultimately increasing the chances of survival and positively impacting recruitment^33^.

Previous studies suggested that enrichment processes and stratification/calm ocean conditions explain both the strength of recruitment success and suitable habitat conditions for small pelagic species. However, these studies tended to focus on the absolute values of the proxies for these phenomena, rather than considering their timing and level of synchrony. Some studies found that the level of primary productivity is negatively correlated with the biomass of anchovy individuals in the first year of life^25^. Other studies, such as Bellido et al.^34^, found a relatively weak, but positive, relationship between CHL and the occurrence of anchovy, suggesting that areas with higher primary productivity were more favorable for anchovy. However, in the Gulf of Lions, Van Beveren et al.^35^ found negative correlations between anchovy landings and the Atlantic Multidecadal Oscillation (AMO). The AMO is an index that is strongly linked with both phyto- and zooplankton communities^36^. Variation in MLD has been associated with the growth of anchovy individuals in the Strait of Sicily^30^, which is promoted by the deepening of the layers. In contrast, MLD is negatively correlated with both the density and occurrence of anchovy eggs in the Bay of Biscay^37^. When using the absolute values of these variables, which are space–time specific for each ecosystem in which anchovy populations occur, it is difficult to explain how environmental-driven fluctuations of anchovy are driven at a regional scale.

Our results were consistent with the existence of strong differences among the three investigated ecosystems in terms of trophic regime, as observed in previous studies^28,32^. By inspecting the temporal behavior of MLD and CHL, two patterns were confirmed in the Mediterranean Sea; specifically, bloom events and high MLD-regulation of CHL in the GoL and AS, and CHL concentrations with a subtropical-like regime in the SoS^28,32^. These regions had distinct trophic regimes supporting different levels of absolute anchovy abundance, with this phenomenon being generally observed for this species and other forage fish e.g.^38,39^. Lower fish abundance occurs in oligotrophic areas (e.g., SoS) versus higher fish abundance in more productive areas (e.g., GoL and AS). MLD patterns regulate access to nutrients and light during phytoplankton growth^28,32^. Thus, based on the synchronicity index, if CHL dynamics follows MLD patterns during the periods spanning anchovy ELS and growth, the abundance of anchovy recruits would be enhanced in all three study areas.

Various authors have demonstrated that rises in population-level fitness occur when the peak of the most energetically demanding period matches that of food availability^40,41^. For example, Kodama et al. 2018 showed that the delay of phytoplankton bloom favored the recruitment of Japanese sardine, specifically the delayed end of bloom improved the larvae feeding environment^42^. Likewise, for haddock inhabiting the continental shelf of Nova Scotia, the timing of local spring blooms favored the survival of ELS^43^, with similar results being obtained for Pacific herring in the Strait of Georgia^44^. In the Gulf of Alaska, warming events increase the risk of prey mismatch for Pacific cod larvae, which affects recruitment, by reducing the time window for the larvae first-feeding and shifting the onset of Chlorophyll-a^45^. Further, for the Northwest Atlantic mackerel, although the stock size supports a long-term average recruitment value, the latter peaked when an optimal temporal and/or spatial match between mackerel larvae and their main prey occurred^46^. Similarly, in the Norwegian-Barents Sea system, the Northeast Arctic cod showed successful recruitment when both the location and timing of high abundances of cod larvae and zooplankton prey overlapped^47^.

Although the approach used in the present study differed slightly from previous analyses, the results were similar in that the importance of the processes of food availability, concentration, and retention were highlighted. MLD regulates CHL phenology physically^28,48^, and is crucial for nutrient concentration and ELS retention processes^30^. Thus, by focusing on synchronicity, rather than the absolute values of CHL and MLD during the critical early life stages of anchovy, we demonstrated a putative spatiotemporal trophic linkage of bottom-up control for areas with different production levels.

Within the three areas over the study period, anchovy recruitment has been subjected to alternating periods of abundance and scarcity. In this study we showed that the synchronicity index effectively and consistently described this recruitment variability by depicting those environmental processes favoring the survival and growth of ELS and juvenile anchovy in different areas of the Mediterranean Sea. Understanding recruitment–environment relationships is a fundamental component of fishery science, with this information being important for enhancing management e.g.^22,49,50^. Better insights on how chlorophyll concentration changes in relation to the mixed layer in both time and space, along with other drivers (such as top-down forcing factors) could further strengthen statistical descriptions of 1-year-age class anchovy. The approach developed here could be easily applied to other ecosystems and used to assess the impact of projected environmental changes. Continued surveys and collection of oceanographic data, which were used in the analyses of this study, would enlarge time series of the dataset, potentially providing insights on the mechanisms regulating fluctuations in recruitment, which is essential for the sustainable exploitation of small pelagic fisheries.

## Methods

### Study areas

The study included three marine areas frequented by European anchovies in the Mediterranean basin; specifically, the Strait of Sicily, the Adriatic Sea, and the Gulf of Lions (Fig. 1).

The Strait of Sicily (SoS) is located between the eastern and western Mediterranean subregions; only the northern part of the SoS was evaluated here, corresponding to the area surveyed by the Mediterranean International Acoustic Survey (MEDIAS). This area is characterized by a narrow continental shelf that widens in both the eastern and western parts. The Atlantic Ionian Stream (AIS) flows eastward through this area, and is the main driver of surface circulation^51^, promoting the formation of two large anticyclonic gyres in the two wide shelves e.g.^52^. The AIS also contributes to the formation of a permanent coastal upwelling, supporting primary production in the area, and is reinforced by wind-induced events^53,54^.

The Adriatic Sea (AS) is located in the central part of the Mediterranean basin, between the Italian and Balkan peninsulas. It is characterized by a wide continental shelf that gently slopes along the major axis (northwest-southeast direction) to the southern part, where a depression more than 1200 m deep occurs. The interaction between strong river runoff from the Po River and other minor rivers (enhancing primary production^55^), with heat loss caused by the Bora wind (northeast direction) influence circulation in the basin^56^. One current branch flows northwards in the eastern part and one flows southwards along the Italian coast, with a cyclonic surface circulation that interacts with the bottom topography in the central and southern part of the basin and contributes to the formation of seasonal cyclonic gyres^57^.

The Gulf of Lions (GoL) is situated in the north-western basin of the Mediterranean Sea, and represents one of the most productive zones in this subregion. This high productivity includes phytoplankton blooms on the continental shelf that expand throughout the whole GoL, due to high river inflow from the Rhone River and upwelling events caused by the two main northerly winds, the Mistral and Tramontane^58,59^.

### Anchovy recruitment and environmental data

For SoS and AD, the time series on the total abundance of anchovy recruits (1-year-old individuals) was obtained from publicly available, validated stock assessment forms (SAFs, http://www.fao.org/gfcm/data/safs, 2019 and 2018 respectively), while for GoL from the Scientific Advisory Committee on Fisheries (SAC, 2021) of the General Fisheries Commission for the Mediterranean (GFCM). Source data were collected in the framework of the MEDIAS and Pelagique Mediteranee surveys (PELMED), which are designed to sample and assess small pelagic fish within the continental shelf during the summer of each year. MEDIAS is carried out in the SoS and the AS during July and June, respectively. PELMED is carried out in the GoL during July. The longest temporal series were obtained from the SAF annual reports of 2019 and 2018 for the SoS (2002–2019, n° years = 18) and AS (2004–2018, n° years = 15) respectively, and from the SAC report for the GoL (2002–2019, n° years = 18).

Mean monthly images of chlorophyll-a (CHL, mg/m^3^), mixed layer depth (MLD, m), and sea surface temperature (SST,°C) were obtained from the Copernicus Marine services model for the Mediterranean Sea (available from http://marine.copernicus.eu/). For both the CHL and MLD series, the spatial average per area of each monthly image was calculated to obtain environmental monthly series characterizing the three study areas separately.

### Synchronicity index

In the middle and high latitudes, the seasonal evolution of phytoplankton concentration and MLD is strongly associated^60^, matching the definition of synchrony (i.e., similar dynamics over time for different components in the system). To describe the strength of the synchrony in variation between CHL and MLD, a robust measure of the Pearson correlation coefficient was used, which guards against the impact of outliers^61^; this measure was subsequently termed the Synchronicity Index (SI). SI is the “*percentage bend correlation”,* which is the Pearson correlation computed on transformed data obtained by down weighting a specific percentage of marginal observations deviating from the median^62^. The 95% confidence interval (CI) of the SI was obtained by resampling pairs of observations with replacement. SI was computed using the WRSE package^63^ in R software (R Core Team, 2019), and a default value of 0.2 was selected (i.e., the proportion of data that could be changed without biasing the estimator) for the percentage bend, in line with Wilcox^61^. The SI for the three separate areas was estimated for each year by correlating CHL and MLD from the month corresponding to the beginning of the spawning season until the month preceding MEDIAS and PELMED observations of the subsequent year. Thus, for each annual anchovy observation, SI was calculated from April (i.e., when anchovy start spawning^10,11^) to June for SoS and GoL, and until May for AS. The higher and more significant the robust correlation estimate, the stronger the synchronicity between the two environmental variables. The same time window that was used to calculate annual SI was used for the average SST monthly series and to characterize the thermal environment of anchovy during the first year of life.

### SI, SST, and relationship with anchovy recruitment

For each area, a preliminary test for the presence of significant associations between annual SI and SST using Pearson correlation was performed and showed no significant relationship in any of the considered areas (ρ: SoS = −0.28^ ns^; AS = −0.44^ ns^; GoL = 0.21^ ns^).

The abundance of the anchovy recruits from each area was standardized by using the min–max scaling. This approach transforms the original data of each stock such that they are within the range [0,1]. Min–max scaling is performed by subtracting to the recruitment the minimum value of the series and dividing this value by the difference between the maximum and the minimum abundance. The same transformation was used for both the SI and the SST of each area. This standardization allowed us to analyze the data altogether to assess the overall effect that the SI and the SST have on recruitment within the Mediterranean Sea. The shape and the strength of the relationship were obtained by using the robust linear regression model^64^. The regression coefficient estimates in this modeling framework are obtained by minimizing the weighted residuals iteratively^64^ and allowed us to reduce the impact of the influential and unusual observations^64,65^. We introduced both the predictors within the model with the standardized abundance, log(x + 1) transformed, as dependent variable. The significance of the parameters was obtained by constructing the first order normal approximation (Normal) interval, the bootstrap percentile interval (Percentile), and the adjusted bootstrap percentile (BCa), from the estimates obtained with the random-X resampling procedure^66^. This bootstrap procedure consists of selecting an N bootstrap sample (n° sample = 2999), fitting the robust regression model, and saving the coefficient to calculate the bootstrap intervals above.

For the model obtained, residuals were checked for normality, homogeneity in variance and independence (Fig. S2).

All analyses were performed using the R statistical software (R Core Team, 2019).

## Supplementary Information

Supplementary Figures.
