# Supplementary material for: Revealing environmental synchronicity that enhances anchovy recruitment in the Mediterranean Sea
Source: Sci Rep. 2022 Apr 4;12:5600. doi: 10.1038/s41598-022-09418-z (PMC8979991; doi:10.1038/s41598-022-09418-z)
Supplement: Supplementary file 1 — Supplementary Figures. [file 41598_2022_9418_MOESM1_ESM.docx]

**Supplementary information**

**for**

**revealing environmental synchronicity that enhances anchovy recruitment in the mediterranean sea**

F. Quattrocchi^1^, G. Garofalo^1^

^1^National Research Council - Institute for Marine Biological Resources and Biotechnology (CNR IRBIM), SS Mazara del Vallo, Via L. Vaccara 61, 91026, Mazara del Vallo (TP), Italy

Corresponding author:

Federico Quattrocchi, federico.quattrocchi@irbim.cnr.it


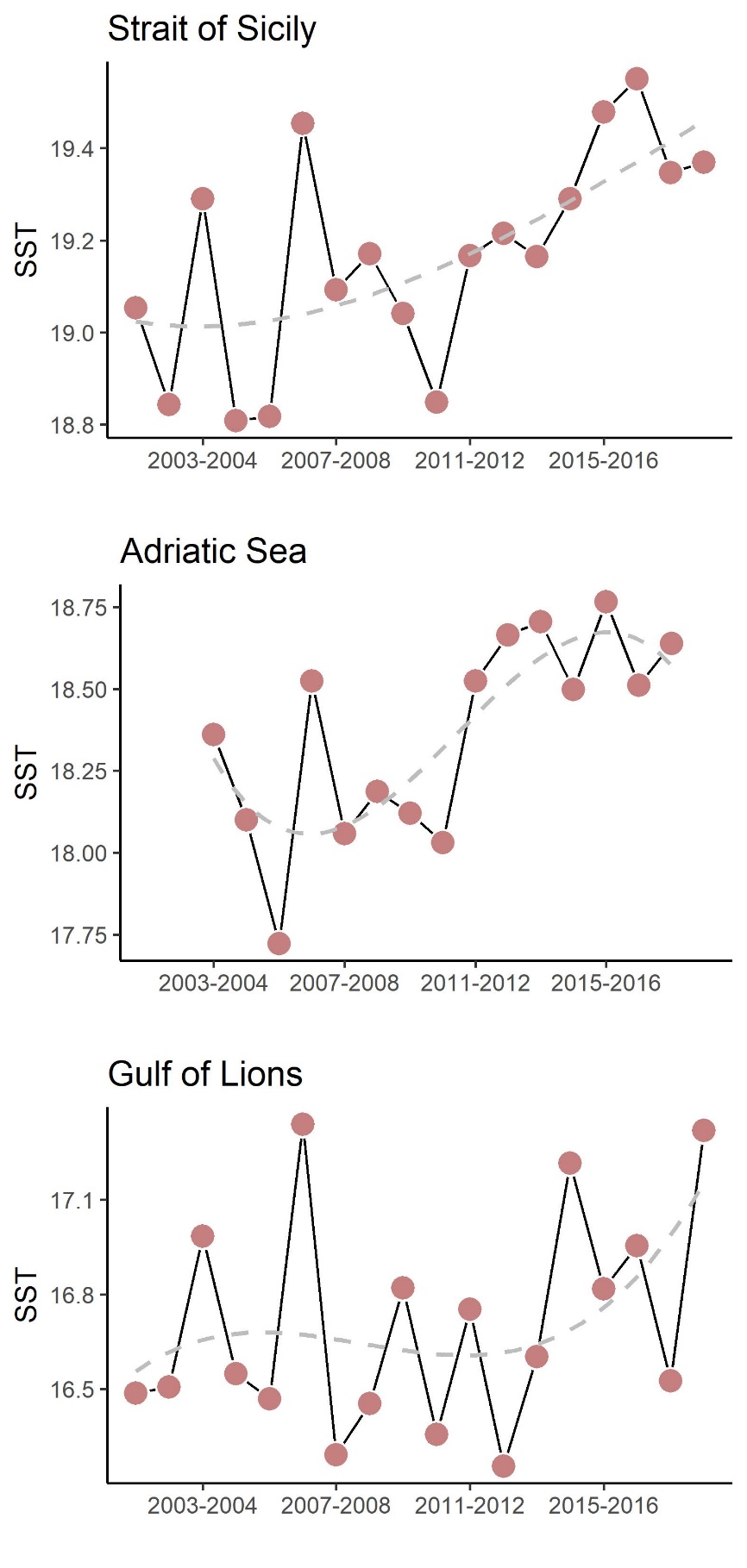


**Figure S1**. Fluctuation in sea surface temperature (SST) in the three ecosystems (Gulf of Lions, Adriatic Sea, and Strait of Sicily).


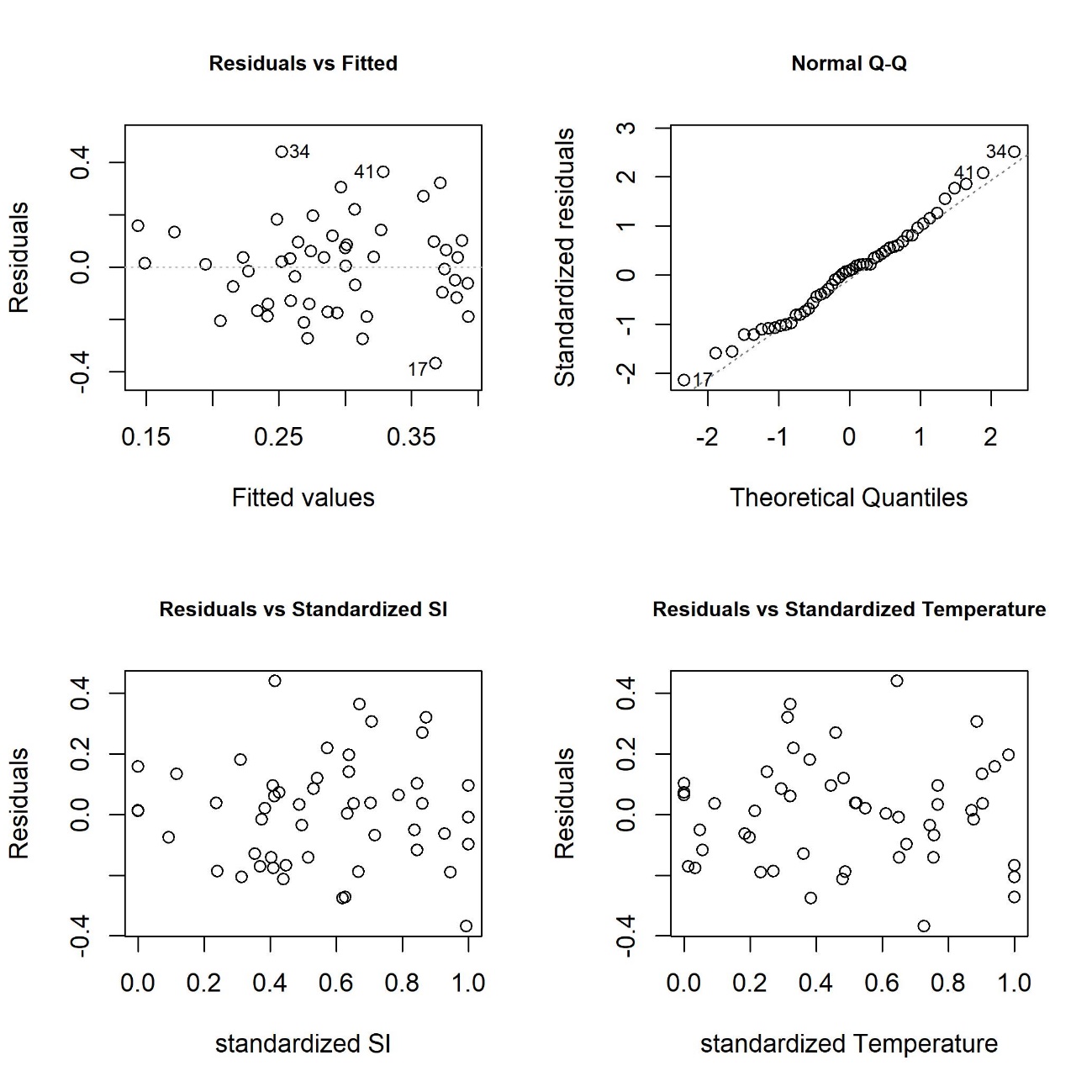


**Figure S2**. Model validation graphs. Top left: Fitted values versus residuals (homogeneity). top right: QQ-plot for (normality). Bottom left: Residuals versus Standardized SI (independence assumption). Bottom right: Residuals versus Standardized SI (independence assumption).
